# Advanced Growth Factor Delivery Systems in Wound Management and Skin Regeneration

**DOI:** 10.3390/molecules22081259

**Published:** 2017-07-27

**Authors:** Jin Woo Park, Seung Rim Hwang, In-Soo Yoon

**Affiliations:** 1 Department of Pharmacy, College of Pharmacy and Natural Medicine Research Institute, Mokpo National University, Muan-gun, Jeonnam 58554, Korea; jwpark@mokpo.ac.kr; 2 Department of Pharmacy, College of Pharmacy, Chosun University, Dong-gu, Gwangju 61452, Korea; srhwang@chosun.ac.kr; 3 College of Pharmacy, Pusan National University, Geumjeong-gu, Busan 46241, Korea

**Keywords:** chronic wound, drug delivery system, growth factor, wound healing

## Abstract

Growth factors are endogenous signaling molecules that regulate cellular responses required for wound healing processes such as migration, proliferation, and differentiation. However, exogenous application of growth factors has limited effectiveness in clinical settings due to their low in vivo stability, restricted absorption through skin around wound lesions, elimination by exudation prior to reaching the wound area, and other unwanted side effects. Sophisticated systems to control the spatio-temporal delivery of growth factors are required for the effective and safe use of growth factors as regenerative treatments in clinical practice, such as biomaterial-based drug delivery systems (DDSs). The current review describes the roles of growth factors in wound healing, their clinical applications for the treatment of chronic wounds, and advances in growth factor-loaded DDSs for enhanced wound healing, focusing on micro- and nano-particulate systems, scaffolds, hydrogels, and other miscellaneous systems.

## 1. Introduction

A chronic wound is defined as a wound that does not heal in an orderly set of phases or in a timely fashion [1]. In recent decades, the number of people suffering from impaired wound healing has increased. Approximately 1–2% of the population of the United States and Europe is affected by chronic wounds, requiring a financial commitment of 2–4% of total health budgets from governments and an average of 6000–10,000 EUR per patient per annum [2,3,4]. Many studies have focused on finding novel therapies for wound healing and skin regeneration.

Growth factors are endogenous signaling molecules that regulate cellular responses for the wound healing processes of migration, proliferation, and differentiation [5]. Growth factors offer promise for optimal wound management as the understanding of their role in the pathophysiology of chronic wounds increases; however, they have limited clinical applications because of a short in vivo half-life due to their low stability, restricted absorption through the skin around wound lesions, elimination by exudation before reaching the wound area, and undesirable effects due to high local and/or systemic levels after topical administration [6]. Wound healing is a complex process influenced by a variety of factors. At each healing stage, a different set of specific cytokines and growth factors must interact with their receptors, other growth factors, and extracellular matrix (ECM) components at their target sites [7]. Sophisticated drug delivery systems (DDSs) are needed to prevent growth factor degradation at the wound site and allow for the spatio-temporally controlled delivery of an appropriate combination of growth factors [8]. Biomaterial-based DDSs are currently in development for the effective and safe use of growth factors as regenerative medicines in clinical practice [9].

This review discusses current advances in growth factor-loaded DDSs for wound healing enhancement, focusing on micro- and nano-particulate systems, scaffolds, and hydrogels. A general overview of the roles of growth factors in wound healing and their clinical applications for chronic wound treatment is also provided.

## 2. Wound Healing and Its Associated Complications

Wound healing is a complex physiological process involving the interplay among various types of cells, growth factors, ECM components, and proteinases [10]. During the normal wound healing process, the phases of inflammation, proliferation, and remodeling occur sequentially in a continuous and sometimes overlapping fashion [11].

### 2.1. Hemostasis and Inflammation

Injury to the skin exposes intravascular platelets to subendothelial collagen, leading to thrombin formation [12]. Platelets activated by thrombin release several growth factors, eventually forming a hemostatic plug [13]. Growth factors released from activated platelets include epidermal growth factor (EGF), heparin-binding EGF-like growth factor, insulin-like growth factor 1 (IGF-1), platelet-derived endothelial cell growth factor, platelet-derived EGF, platelet-derived growth factor (PDGF), transforming growth factor (TGF)-alpha and TGF-beta (TGF-β) [11,14]. These growth factors diffuse into surrounding tissues and chemotactically attract neutrophils and monocytes into the wound [15]. The monocytes differentiate into macrophages, which mediate a series of processes vital for normal wound healing [16,17]. This acute inflammatory process lasts 1‒2 days in uncomplicated wounds [15].

### 2.2. Proliferation (Granulation Tissue Formation)

The proliferation phase is characterized by the formation of granulation tissue and initiation of angiogenesis [18]. Granulation tissue is composed of fibroblasts, neovasculature, and macrophages in a loose matrix of collagen, hyaluronic acid (HA), and fibronectin, and it eventually fills the wound area [19]. The number of inflammatory cells is reduced during this phase, and PDGF and TGF-β released from inflammatory cells chemotactically attract fibroblasts into the wound area [20]. Macrophages provide a continual source of growth factors, including fibroblast growth factor (FGF), which induces the activation and proliferation of fibroblasts that later produce the ECM [21]. Fibroblast migration and proliferation occur during the initial 2‒3 days post-injury. The fibroblasts then release collagen and glycosaminoglycans, such as chondroitin-4-sulphate, dermatan sulfate, heparin sulfate, and HA [22], which form an amorphous gel in which collagen fibers are deposited and then aggregate. Collagen and fibronectin form the ECM, essential for formation of granulation tissue [18]. Matrix metalloproteinases (MMPs) can eliminate fibers not required to increase the structural integrity of the wound. MMPs and their inhibitors (tissue inhibitors of matrix metalloproteinases; TIMPs) are derived from fibroblasts, and their final outcome in the wound environment is the net effect of their opposing activities [11]. Fibroblast proliferation occurs together with angiogenesis, which allows nutrients and wound-healing factors to enter the wound area [12]. Growth factors primarily responsible for regulating angiogenesis include vascular endothelial growth factor (VEGF) released from macrophages or keratinocytes, and basic FGF (bFGF) released from macrophages or damaged endothelial cells [23].

### 2.3. Remodeling (Maturation)

Maturation is the final phase of wound healing and can last from 3 weeks to 2 years post-injury [24]. Fibroblasts begin to decrease as collagen is deposited [21]. In contrast to intact skin, which is predominantly comprised of type I and III collagen [22], granulation tissue has a higher type III collagen content, and the newly formed collagen fibers in the wound are unorganized and arranged randomly [25]. As wound healing progresses, type III collagen is gradually replaced with type I collagen, and the collagen fibers are rearranged into a more organized lattice structure, increasing the mechanical strength of the tissue [11], although newly formed scar tissue has only 70‒80% of the tensile strength of intact skin [15].

### 2.4. Complications in Wound Healing

The major pathologies of wound healing include chronic wounds and scarring. A chronic wound refers to a wound that does not heal in an orderly set of phases or wound healing that deviates from the norm [11]. A number of pathophysiological factors can cause the failure of normal wound healing, including inflammation, infection, malnutrition, age, diabetes, tissue maceration, pressure necrosis, and renal impairment [1,26,27]. Diabetic foot ulcers (DFUs) are a relatively common form of chronic wound; approximately 20% of all diabetic patients experience one in their lifetime [3,4]. DFUs are primarily caused by peripheral neuropathy, both sensory and motor, as well as vascular disease coupled with an unrecognized repetitive minor trauma due to high glucose levels [28]. Ulceration often occurs due to the loss of pain sensation, which results in severe infection or peripheral ischemia and can lead to amputation [29]. Chronic wound treatments include moisturizing the wound area, controlling bacterial load, and/or debriding the necrotic tissue [1]. These therapies cannot guarantee complete healing without recurrence because the chronic wound healing process can take several days, weeks, or months and is characterized by an extended inflammatory phase, delayed cellular proliferation, and poor re-epithelialization and remodeling with impaired angiogenesis [30,31]. One of the most common chronic wound complications is that of elevated levels of inflammatory cells that secrete matrix metalloproteinases (MMPs) and other proteases, which result in a deficiency of cytokines, especially the growth factors that provide the cellular and molecular signals necessary for normal healing [32]. To be successful, active treatment must convert the wound environment from a chronic to an acute state [11,33].

A scar is a densely bundled orientated collagen fibrous tissue that replaces normal tissue after injury. The two types of scars in human skin are hypertrophic and keloid scars [34]. Both scar types are caused by an overproduction of immature collagen during the remodeling phase. However, hypertrophic scars form a red raised lump on the original wound area, which can partially regress over time, while keloid scars (a more serious form of excessive scarring) extend beyond the original wound area with thicker collagen bundles, which do not regress spontaneously [35]. Several treatment regimens for these scars have been developed, including corticosteroids, cryotherapy, radiation, laser therapy, pressure therapy, and silicone gel sheeting, but none are optimal [36]. Although the exact molecular mechanism of scarring remains unclear, it is believed that common signaling molecules including TGF-β, PDGF, IL-4/13, connective tissue growth factor, and osteopontin are involved in the fibrosis that leads to scarring [34].

## 3. Roles of Growth Factors in Wound Healing and Current Clinical Applications

### 3.1. Growth Factors Involved in the Wound Healing Process

**Figure 1:**
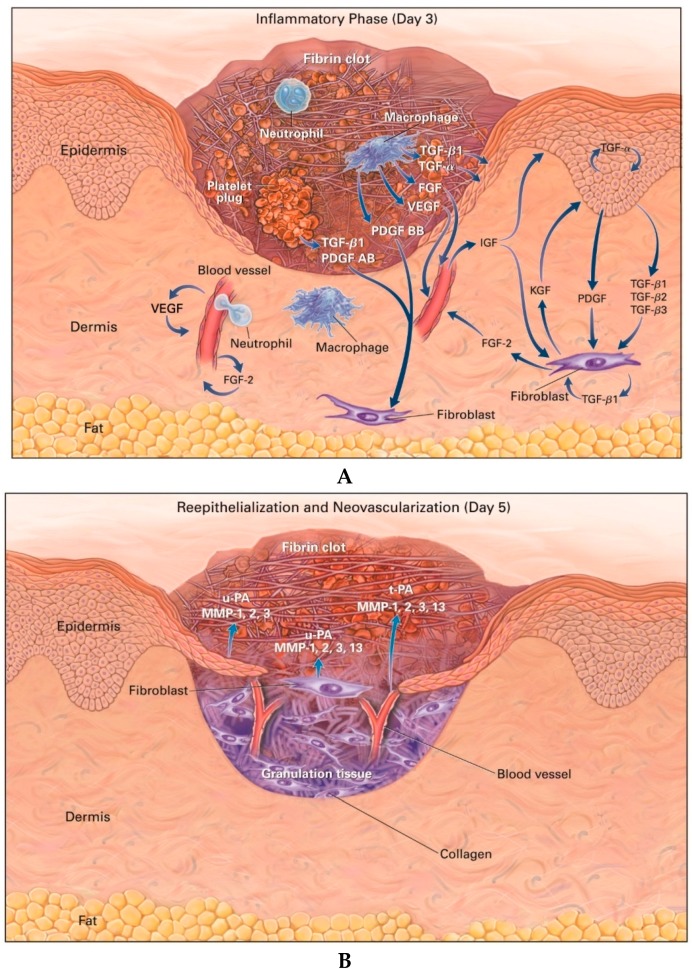
Cutaneous wounds 3 (**A**) and 5 days (**B**) after injury. Growth factors such as fibroblast growth factor (FGF), insulin-like growth factor (IGF), keratinocyte growth factor (KGF), platelet-derived growth factor (PDGF), transforming growth factor (TGF), and vascular endothelial growth factor (VEGF), and proteases such as matrix metalloproteinases (MMPs) and plasminogen activator (PA) are thought to be necessary for cell movement [reproduced with permission from Singer, A.J.; Clark, R.A. Cutaneous wound healing. *N. Engl. J. Med.* **1999**, 341, 738‒746].

Growth factors are naturally occurring polypeptides involved in cell growth, proliferation, migration, and differentiation [37]. The specific binding of a growth factor to its receptor activates intracellular signal transduction pathways that regulate various aspects of subcellular physiology and cellular function [38]. As shown in Figure 1, growth factors bind to their corresponding receptors located on the cell surface, initiating signaling pathways to activate relevant signaling molecules that can activate cytoplasmic proteins or induce transcription of new proteins [21]. Depending on conditions, such as cell type and microenvironment, the same growth factor and receptor can activate different signal transduction pathways and exhibit different cellular responses [38]. Table 1 summarizes the cell sources and main functions of the growth factors involved in wound healing and skin regeneration. Growth factors play a critical role in modulating inflammatory responses, enhancing granulation tissue formation, and inducing angiogenesis. They are essential for successful matrix formation and remodeling processes in the normal wound healing process. Growth factor deficiencies, including reduced levels of bFGF, PDGF, EGF, and TGF-β, have been reported in chronic pressure ulcers when compared with acute wounds [39], and expression of PDGF is shown to be lower in chronic dermal ulcers than in surgically created acute wounds [40]. This suggests that growth factor deficiencies are responsible for chronic wounds [41,42]. Because the functions of growth factors are known to be dependent on their spatial distribution [43], controlling the delivery of growth factors both spatially and temporally is crucial for their effective and safe use as regenerative medicines in clinical practice.

### 3.2. Current Applications of Growth Factors for Wound Healing and Skin Regeneration in Clinical Settings

Topical administration of growth factors is a promising strategy to promote wound healing because of the role of growth factors in wound healing and their deficiency in chronic wounds [46]. Several approved medications that include growth factors are available as preparations for external use in the form of solutions, gels, creams, and ointments. Clinical trials of topically administered growth factors have shown conflicting evidence for therapeutic outcomes [53]. Regranex^®^ Gel is an aqueous-based sodium carboxymethylcellulose gel containing 0.01% becaplermin, a recombinant human PDGF (rhPDGF) approved for topical use by the US Food and Drug Administration (FDA). It is indicated for the treatment of chronic DFUs, when used as an adjuvant with appropriate ulcer care practices such as initial sharp debridement, pressure relief, and infection control [2,54]. In one study, treatment with Regranex^®^ gel was approximately twice as effective as appropriate ulcer care alone or placebo gel [55], while another study showed no significant difference in the efficacy of good ulcer care with and without becaplermin gel [56]. The rhPDGF gel also has off-label uses for acute full-thickness wounds. A double-blind controlled study showed that application of the rhPDGF gel increased the rate of acute wound healing [57]. Fiblast^®^ Spray is a commercially available recombinant human bFGF (rhbFGF) product that is indicated for decubitus and skin ulcers including burn and leg ulcers [58]. Clinical use of rhbFGF solution for DFU treatment was first approved in Japan, where a deep diabetic foot wound was successfully reconstructed with artificial dermis and rhbFGF [59]. However, another double-blind controlled study reported no significant effect of topical rhbFGF application on DFUs, possibly attributable to its low in vivo stability [60,61]. Commercially available medications containing recombinant human EGF (rhEGF) include Heberprot-P^®^, Regen-D™ 150, and Easyef^®^. Heberprot-P^®^ contains 75 μg of freeze-dried rhEGF and is administered intralesionally three times per week. It is intended for treatment of DFUs to avoid lower limb amputations. A study of 20 diabetic patients given intralesional injections of 75 μg Heberprot-P three times per week showed full granulation response in all patients, complete wound closure in 17 patients, and no lower limb amputations [62]. Regen-D™ 150, commercialized in India for DFU treatment, is a gel containing 150 μg/g rhEGF that is applied topically twice daily until complete healing [47]. Easyef^®^ is a dermal solution spray indicated for the treatment of DFUs. An open-label trial, crossover, and prospective study reported that 21 of 89 patients showed improvement without rhEGF treatment, while complete healing of chronic DFUs was observed in 52 of 68 patients treated with rhEGF [63].

Wound healing is a complex process influenced by a variety of different factors. At each stage of healing, a different set of specific cytokines and growth factors are required [64], while several proteases activated in the wound area can degrade both endogenous and exogenous growth factors [65]. Topically administered growth factors in chronic wounds have shown limited success because of the difficulty associated with permeation through the outermost skin layer surrounding the lesion. The strong barrier function of the stratum corneum to hydrophilic macromolecules, as well as rapid elimination by exudation in the wound bed, limit the efficacy of growth factor topical applications [66]. Conventional formulation strategies may not provide the required residence time for growth factors in the wound area to interact with the target cells, due to their high degradation rate [67]. Current medications containing growth factors require high doses and/or repeated administration over a long period of time, which can lead to serious side effects including oncogenesis. Such supra-physiological dosing of growth factors also increases the cost of the therapy [38]. Growth factor delivery systems that improve the stability of growth factors in the wound area and control the release of growth factors provide more effective and safe treatment options.

### 3.3. Platelet-Rich Plasma Therapy for Advanced Wound Management

As mentioned above, wound healing is a dynamic and complex process, and no single exogenous agent can effectively facilitate all aspects of the wound-healing response [68]. Therefore, a combination therapy is required for successful cutaneous wound repair, and platelets have been used as a rich source of growth factors [69].

During the natural wound healing process, platelets are one of the first cell types to respond at the wound site, playing a pivotal role in the initiation of wound healing [70]. After activation and degranulation by thrombin, platelets release the α-granule contents, including potent mitogenic and chemotactic factors that are important in wound healing, such as coagulation factors, fibrinogen, platelet thromboplastin, thrombospondin, PDGF, TGF-β, VEGF, EGF, IGF, calcium, serotonin, histamine, and hydrolytic enzymes [71,72,73,74]. Therefore, administration of autologous platelet-rich plasma (PRP) gel on the wound sites has been proposed as a novel strategy to promote the wound-healing cascade and tissue regeneration in chronic and non-healing wounds, as well as soft tissue ulcerations [75,76,77,78].

PRP is defined as a portion of the plasma fraction with a high concentration of autologous platelets (at least one million per microliter plasma) [79,80]. Commonly, PRP is used in a gel formulation prepared via a two-step process: centrifugation of autologous whole blood to separate the plasma from packed red blood cells followed by centrifugation to separate PRP from platelet-poor plasma. The concentrate is further activated via the addition of thrombin or calcium, resulting in a platelet gel [74,81]. The AutoloGel™ System (Cytomedix, Inc., Rockville, MD, USA), which contains all materials including bovine thrombin, is the autologous PRP separation system currently indicated for use in diabetic ulcers [74,82].

The PRP gel provides more similarity to the natural wound healing process involving multiple growth factors in their biologically determined ratios. In addition, the PRP gel acts as a tissue sealant and sustained delivery system for accelerating bone repair, promoting fibroblast proliferation, and increasing tissue vascularity by releasing growth factors more effectively and directly via the degranulation of α-granules [74,80]. However, PRP therapy is considered a more cost-effective and economical use of resources for the treatment of DFUs, with no special considerations of antibody formation or risk of donor disease [83]. At this time, the promising indications for PRP include acceleration of healing in all surgical fields, such as periodontal and oral, maxillofacial, orthopedic and trauma, aesthetic, spinal, and heart bypass surgeries, as well as treatment of acute and chronic non-healing wounds in diabetic patients and severe burns and associated skin grafts [76,77,84,85,86,87,88]. Based on a meta-analysis of PRP therapy in acute and chronic wound studies, complete and partial wound healing during PRP treatment was improved compared with the control wound care [89]. Driver et al. performed the first reported prospective, randomized, controlled, multicenter trial in the United States regarding the use of autologous PRP for the treatment of 72 patients with DFUs; they reported that excluding 32 patients, 68.4% in the PRP treatment group and 42.9% in the control group healed after 12 weeks, and wounds in the PRP group were repaired after a mean of 42.9 days (47.4 days in the control group) [90].

Although many studies showed that the application of PRP therapy favored complete wound healing compared with control care, large prospective and randomized trials using standardized protocols, including the absolute concentrations and activation rates of platelets, growth factor profiles, and the number of applications are required to expand its clinical use.

## 4. Growth Factor Delivery Systems

**Figure 2:**
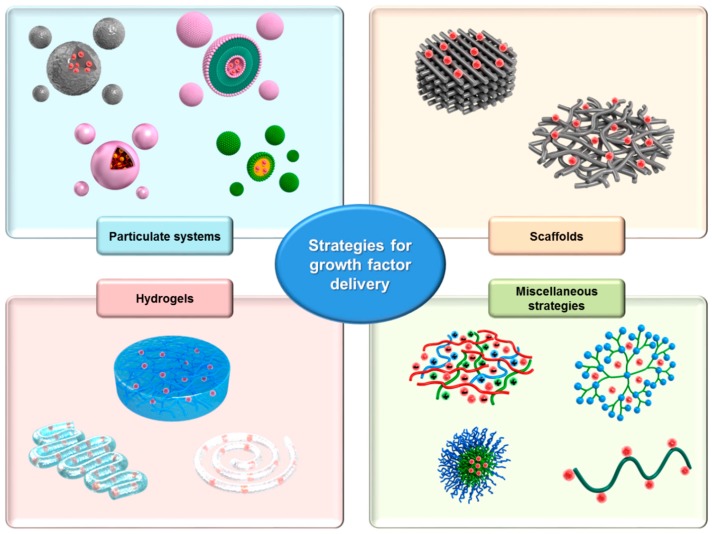
Schematic illustration of growth factor-loaded drug delivery systems (DDSs) for enhanced wound healing. DDSs were classified according to their matrix structures, i.e., particulate systems, scaffolds, hydrogels, and miscellaneous strategies.

Several different DDSs have been developed to provide improved stability and controlled release of growth factors for the treatment of acute and chronic wounds. In the sections below, DDSs for growth factors are classified according to their matrix structures, i.e., particulates, scaffolds, hydrogels, and miscellaneous systems. A schematic illustration of the DDS classifications described in this section is shown in Figure 2. For each classification, the drug loading procedures, administration routes, dosing regimens, and therapeutic results for specific DDSs are described in detail. A brief summary of the DDSs described in this section is also provided in Table 2.

### 4.1. Particulate Systems

In contrast to other types of DDSs, particulate DDSs are characterized by their size, i.e., 1–1000 μm for microparticles (microspheres) and <1000 nm for nanoparticles (nanospheres) [118]. The structural and physicochemical properties of nanoparticulates lead to improved stability, liberation, bioavailability, biodistribution, and immunogenicity in vivo of loaded drugs, leading to more efficient delivery of growth factors [38]. Among the various nanoparticulate systems, lipid nanoparticles have been developed for dermal applications because of their excellent biocompatibility [119]. For example, liposomes, which are spherical vesicles consisting of an aqueous solution core surrounded by one or more phospholipid bilayers, have been used clinically for drug delivery [120,121]. Hydrophilic solutes dissolved in the core and hydrophobic substances associated with the bilayer give liposomes their amphiphilic characteristics [122]. Alemdaroğlu et al. found that EGF-loaded multilamellar liposomes, prepared by film formation, exhibited healing effects on second-degree burn wounds in rats [91]. After 14 days, healing effects were found to be highest in rats treated with EGF-liposome, followed by those treated with Silverdine ointment, and finally, those treated with EGF solution without liposome. This suggests that the EGF-liposome formulation may be effective for burn wound healing. In a study of rats subjected to 40% full-thickness scald injury, Pierre et al. reported that a small dose of IGF-1 loaded in liposomes (0.9 μg/kg/week, subcutaneous injection) was equivalently effective in promoting burn wound re-epithelialization to a higher dose of IGF-1 solution (5.0 mg/kg/day, subcutaneous injection) with growth hormones and more effective than IGF-1 without growth hormones [123]. There are several limitations to the use of liposomes, such as low reproducibility, low stability with the associated burst release, heterogeneous size distribution, and low loading capacity [124]. To overcome these limitations, nanoparticle systems such as solid lipid nanoparticles (SLNs) and nanostructured lipid carriers (NLCs) have been developed. SLNs and NLCs differ in the composition and structure of their lipid core matrices; SLNs are prepared using solid lipids only, while NLCs (that are solid at body temperature) are prepared by mixing solid and liquid (oil) lipids [125,126]. Gainza et al. developed rhEGF-loaded SLNs and NLCs using an emulsification-ultrasonication method; topical administration of the nanoparticulate formulations on full-thickness wounds in genetically diabetic (db/db) mice significantly improved wound closure, restoration of inflammation, and re-epithelization [92]. More recently, rhEGF-loaded NLCs also enhanced wound closure and the ratio of healed wounds in a porcine full-thickness excisional wound model, which is considered a more relevant preclinical model of wound healing [93].

Biodegradable polymers, widely used in drug delivery, can be applied as a wound dressing to inhibit wound contraction and stimulate the healing process [127]. Polymeric micro/nano particulates prepared with biodegradable synthetic or natural polymers, such as poly(lactic-co-glycolic acid) (PLGA), alginate, and gelatin, have been used to deliver growth factors [128]. For example, Dong et al. utilized rhEGF-loaded PLGA microspheres prepared by water-in-oil-in-water (W/O/W) extraction-evaporation to treat chronic diabetic ulcers in rats [94]. Compared to treatment with pure rhEGF, the microspheres increased fibroblast proliferation in vitro and improved wound healing in vivo. Additionally, the microspheres increased the expression of proliferating cell nuclear antigen in the epidermis layer. Chu et al. prepared rhEGF-loaded PLGA nanoparticles using a modified double-emulsion method, and achieved a high encapsulation efficiency of 85.6% [95]. The rhEGF nanoparticles were found to promote fibroblast proliferation and enhance healing rates in diabetic rats with full-thickness wounds. In a similar study, Gainza et al. prepared rhEGF-loaded PLGA-alginate microspheres using a modified W/O/W double-emulsion/solvent evaporation method [96]. The incorporation of alginate improved the encapsulation efficiency and particle size distribution. Diabetic Wistar rats with full-thickness wounds treated with the rhEGF microspheres exhibited significantly reduced wound areas at days 7 and 11, a complete re-epithelization by day 11, and an earlier termination of the inflammatory process. With regard to natural polymer-based particulate systems, Elcin et al. developed VEGF-loaded alginate microspheres prepared using an ion exchange method [97]. The in vitro analyses indicated that the microspheres exhibited a very low initial VEGF release profile up to day 3 and uncontrolled release during days 4 to 7; thereafter, zero-order VEGF release profiles were observed at rates of 50‒90 and 70‒120 ng/day from microspheres containing 2 and 4 μg VEGF/cm^3^, respectively. The 4 μg VEGF-loaded microspheres implanted in the groin of Wistar rats produced a significantly higher level of capillary network formation than the 2 μg VEGF-loaded and the control (empty) microspheres.

### 4.2. Scaffolds

Scaffolds can serve as a 3-dimensional supporting structure for cell growth and differentiation as well as a platform for growth factor delivery, potentially leading to more efficient wound healing. Growth factors are loaded onto scaffolds by physical adsorption or encapsulation during scaffold preparation processes such as phase separation, particulate leaching and solvent casting, gas foaming, freeze drying, and melt molding [38]. The growth factor-loaded scaffold is implanted at the wound site to protect the tissue defect and promote wound healing. Several growth factor-loaded scaffolds have been prepared using biomaterials derived from native ECM, such as HA, collagen, and chitosan. A Kitasato University research group published several papers discussing the preparation of HA- and/or collagen-based sponge-type scaffolds containing EGF or bFGF and their application as a wound dressing [98,99,100,129,130,131]. Topical application of the scaffolds were found to promote wound healing by enhancing cell migration and proliferation, granulation tissue formation, re-epithelization, and angiogenesis. With regard to chitosan, Mizuno et al. developed bFGF-loaded chitosan film prepared by freeze-drying the mixture of bFGF solution and hydroxypropylchitosan (a water-soluble derivative of chitosan) [60]. Activity of bFGF in the chitosan film remained stable at 5 °C for the 21-day study period. The in vivo analysis in genetically diabetic mice revealed an enhanced wound contraction rate and increased granulation tissue formation following application of bFGF-loaded chitosan film compared with the chitosan film alone. Hong et al. studied the effect of rhEGF-loaded chitosan film on the treatment of full-thickness porcine wounds [101]. Continuous release of rhEGF from the chitosan film was found to promote epithelialization; however, no significant changes were observed in the healing time or immunohistochemical staining results.

To better mimic the 3-dimensional porous topology of natural ECM, nanotechnology has been applied to the scaffold preparation processes. For example, micro/nanofibrous structured biomimetic strategies have recently been developed for wound healing enhancement [132]. Micro/nanofibers are fibers with diameters in the micro/nanoscale and generally fabricated using the electrospinning technique. The electrospinning draws nanometric continuous fibers from a polymer solution using an electric charge [133]. It is widely recognized that nanofibers fabricated by this technique mimic the characteristics and architecture of native ECM [102]. Silk, a natural protein fiber obtained from the cocoons of the *Bombyx mori* silkworm, attracts much interest due to its biocompatibility and high mechanical strength [134]. Gil et al. developed EGF-loaded electrospun silk nanofibers to promote wound healing [103]. BALB/c mice treated with the functionalized silk nanofibrous wound dressings showed increased wound healing rates, collagen synthesis, re-epithelization, dermis proliferation, and reduced scar formation, when compared with air-permeable Tegaderm^®^ tape (negative control) or Tegaderm^®^ hydrocolloid dressing (positive control). Recently, Garcia-Orue et al. developed a rhEGF-loaded PLGA nanofibrous membrane prepared by electrospinning and studied its application as a wound dressing [104]. The membrane had fibrous structures with a mean diameter of 356.03 ± 112.05 nm and a porosity of 87.92 ± 11.96%. The membrane improved in vitro fibroblast proliferation as well as promoted significant re-epithelization and wound closure in db/db mice with full-thickness wounds. Nanofibers can be fabricated using two or more natural or synthetic polymers. The combinations of gelatin with poly(l-lactic acid)-co-poly-(ε-caprolactone) (PLLCL) [105] and poly(ε-caprolactone) with poly(ethylene glycol) (PEG) [106] have been used to fabricate EGF-loaded nanofibers and shown to promote wound healing process.

### 4.3. Hydrogels

As described earlier, scaffolds containing growth factors can be extremely useful for wound healing and skin regeneration. However, scaffolds need to be implanted, which may become complicated depending on the morphology of the defect. Hydrogels attract increasing interest because they can be injected and, due to their excellent flexibility, take the shape of the wound site once injected. Hydrogels have a high water content and can thus hold moisture at the wound site; this offers ideal conditions for skin hydration, healing, and removing necrotic tissue. Various natural and synthetic polymers have been used to prepare hydrogels containing growth factors for the treatment of skin defects [135]. The first topical growth factor preparation approved by the FDA (Regranex^®^) was a carboxymethylcellulose hydrogel containing PDGF [107,136]. Carboxymethylcellulose is non-toxic and generally considered to be hypoallergenic; its high viscosity allows for sustained drug release [137]. In clinical trials, the hydrogel was well tolerated and enhanced the healing rate in patients with lower extremity diabetic neuropathic ulcers and decubitus sores. However, the repeated and prolonged use of Regranex^®^ is not recommended by the FDA because of the associated increased risk of cancer [46].

Chitosan is a positively-charged, biodegradable linear polysaccharide that possesses mucoadhesive, coagulation, and antimicrobial properties [138,139]. These characteristics make chitosan hydrogel embedded growth factors promising therapeutics for wound healing. In this regard, Alemdaroğlu et al. utilized an EGF-loaded chitosan gel formulation in the treatment of burn wounds in rats [109]. The immunohistochemical analysis revealed an increase in cell proliferation in rats treated with the EGF-chitosan gel and the EGF solution alone, relative to the control group (without EGF). The histochemical analysis showed an increased epithelization rate in the EGF-chitosan gel-treated group compared to the EGF solution-treated group. Further, chitosan gel formulations containing EGF and egg yolk oil (EYO) have also been found to improve wound healing, relative to Silverdin (1% silver sulfadiazine), in Wistar rats with dermal burns [108].

HA is a component of all connective tissues; it can serve as a potential delivery vehicle for growth factors as well as promote cellular responses to growth factors [140]. Xie et al. employed HA gel as a potential vehicle for vitronectin, IGF, and EGF [140]. The HA gel promoted proliferation and differentiation of epithelial cells in a 3-dimensional de-epidermized-dermis human skin equivalent model, when compared to the active ingredients alone. Considering the low stability of vitronectin and the other growth factors, the HA gel may have improved the in vivo stability of these active ingredients, compared with the administration of their free forms [140].

With regard to other polymers, Suzuki et al. developed cross-linked gelatin gels containing growth factors extracted from platelet-rich fibrin [110]. After 2 weeks of application in rats with full-thickness skin defects, the gel was more effective in promoting wound healing than the commonly used platelet-rich plasma. Liu et al. prepared bFGF-loaded hydrogel films composed of co-crosslinked thiolated derivatives of chondroitin 6-sulfate and heparin, and studied its effect on full-thickness wounds in db/db mice [111]. After a 2-week application, wound closure was 89% in mice treated with the bFGF-loaded hydrogel films and 27% in the controls. Choi and Yoo developed wound-adhesive and thermo-responsive Pluronic-127/chitosan hydrogels for delivery of rhEGF [112]. Pluronic F-127 transforms from an aqueous solution to a hydrogel as the temperature increases from 4 °C to 23 °C [141]. Choi and Yoo found that increasing the blend ratio of chitosan in the hydrogel reduced the release rate of rhEGF and enhanced its mucoadhesive properties. In the treatment of burn wounds in mice, the rhEGF-loaded hydrogel improved healing rates and epidermis regeneration. Goh et al. reported that hydrogel sheets composed of heparin and diacrylated PEG led to sustained release of human EGF in vitro and accelerated wound closure in vivo [119].

### 4.4. Miscellaneous Strategies

In addition to micro/nano-particulate systems, scaffolds, and hydrogels, there are other miscellaneous delivery strategies to improve the transdermal delivery of growth factor for wound healing. To overcome the limitations of particulate-based delivery systems, a coacervate integrating positively charged poly(ethylene argininylaspartate digylceride (PEAD) with negatively charged heparin was developed to stabilize bFGF and prevent burst release [113]. Meanwhile, conjugates between aldehyde-modified HA and the amine group of EGF in the HA film were found to extend the residence time of EGF in the wound area and significantly regenerate skin tissues [114]. The fusion protein of the transactivator of transcription protein and acidic FGF (aFGF) showed efficient penetration of aFGF in skin and enhanced wound healing in deep tissue injury [115]. Additionally, low-molecular-weight protamine can be conjugated to the N-terminal sequence of EGF to enhance EGF penetration without reducing its biological activity [116,117]. Besides single growth factor therapy, the effect of recombinant growth factor mixtures on the proliferation and migration of human skin fibroblasts, expression of cell cycle regulatory proteins or type I collagen, and acceleration of wound healing has been evaluated in animal models [142]. Combination therapy of deciduous teeth dental pulp cell transplantation and bFGF solution has also been examined for wound healing of skin defects in nude mice [143].

Previous studies have explored delivery systems for compounds that can control fibrotic responses to growth factors to reduce the formation of aesthetically disturbing scars. A previous study investigated a target-seeking antifibrotic compound for scar prevention, which consists of a targeting peptide (to recognize angiogenic blood vessels and extravasates into the skin injury) fused with decorin (to prevent excessive fibrosis by inhibiting TGF-β activity) [144]. The recombinant decorin substantially enhanced the neutralizing activity against TGF-β1, selectively accumulated in wounds, promoted wound healing, and suppressed scar formation at doses at which untargeted decorin was inactive [144]. More recently, Shan et al. developed a silk fibroin/gelatin electrospun nanofibrous dressing functionalized with astragaloside IV, a promising agent promoting burn wound healing and preventing scar formation [145]. The astragaloside IV-loaded nanofibrous dressing promoted cell adhesion, proliferation, and wound healing and inhibited scar formation by stimulating wound closure, increasing angiogenesis, and improving collagen organization [145].

Although growth factors mainly control interactions among cells or those between cells and the ECM, therapy involving single growth factor cannot completely manage the complex wound healing process, which is coordinated by the actions of multiple cell types such as fibroblasts, keratinocytes, platelets, and stem cells. Transplantation of skin fibroblasts into diabetic sheep with excisional wounds significantly increased the number of blood vessels and accelerated wound closure [146]. Cultured allogenic keratinocytes provided clinical benefits for patients with venous ulcers or extensive burns [147,148]. Keratinocytes in epidermal substitutes produce IL-1α and TNF-α, which synergistically mediate the secretion of wound-healing factors from fibroblasts in dermal substitutes [149]. A bilayered living cellular construct containing both keratinocytes and fibroblasts showed higher expression of cytokines and growth factors and greater endothelial network formation than did constructs containing only keratinocytes or fibroblasts [150].

Meanwhile, since stem cells can differentiate into specialized cells and self-renew via tissue homeostasis, they play a role in wound healing [151]. Allogenic mesenchymal stem cells transplanted into beagle dogs with cutaneous wounds increased collagen production and cellular proliferation, as well as decreased mRNA expression of IL-2, IFN-γ, bFGF and MMP-2 [152]. Injection of peripheral blood stem cells enhanced the healing of chronic wounds in horses, which were unresponsive to conventional therapies [153]. Treatment using side-population hematopoietic stem cells, well-characterized self-renewing cells, accelerated wound closure in diabetic mice [154]. Compared with bone marrow-derived mesenchymal stem cells, adipose-derived stem cells (ASCs) are easier to isolate and more affordable as an alternative to multipotent cells. Although dermal fibroblasts accelerated wound closure, human ASCs also showed wound-healing effects in nude mice [155].

However, several issues should be considered in the use of cellular therapies, such as the age of the donor, the optimal dose or treatment time, and administration route of the cells [156,157]. Further studies are required to evaluate the complicated effects of cellular therapies, especially those combined with growth factor delivery.

## 5. Conclusions

A promising strategy in the treatment of chronic wounds is the use of biomaterials and growth factors that replicate the microenvironment and activate crucial regenerative pathways conducive to wound healing. A number of researchers have demonstrated that controlling the release of growth factors from pharmaceutical preparations by DDS-based strategies can enhance wound healing and skin regeneration. However, issues regarding safety and cost of the growth factor-loaded DDS preparation in clinical settings should be properly addressed. In particular, robust nonclinical models predicting the safety and pharmacokinetics of growth factors need to be developed. In addition, the use of a single growth factor may be insufficient for optimal wound healing, because several growth factors are involved in different stages of the wound healing process. Thus, sophisticated growth factor delivery systems that enable controlled spatio-temporal delivery need to be developed by finding novel functional biomaterials and/or by combining two or more DDSs for enhanced and prolonged action, with the ultimate aim of mimicking the synergistic wound healing activity of the combinational release profiles of growth factors and ECM components that occurs in real physiological situations. Therefore, in the current situation, an interdisciplinary approach involving pharmaceutical scientists, pathologists, reconstructive surgeons, and engineers needs to be taken in order to develop novel DDS-based therapies in clinical settings. These delivery systems will undoubtedly provide more effective and safe growth factor formulations for wound healing as well as great benefits to patients in the near future.

## Figures and Tables

**Table 1:** Growth factors involved in wound healing and skin regeneration.

Growth Factor	Cell Source	Primary Action in Wound Healing	Ref.
PDGF family			
PDGF	Platelets Fibroblasts Macrophages Vascular endothelial cells Vascular smooth muscle cells	Chemotactically attracts fibroblasts, neutrophils, monocytes, and smooth muscle cells to the wound Activates macrophages to release growth factors Promotes fibroblast proliferation and production of extracellular matrix	[37] [44]
VEGF	Platelets Fibroblasts Macrophages Keratinocytes	Stimulates (lymph)angiogenesis Enhances endothelial cell migration and proliferation	[37] [44] [45]
EGF family			
EGF	Platelets Fibroblasts Macrophages	Stimulates the proliferation of keratinocytes, fibroblasts, vascular endothelial cells Enhances the production of fibronectin	[46] [47]
TGF-α	Platelets Macrophages Keratinocytes	Similar to EGF Induces angiogenesis	[12]
IGF family			
IGF	Fibroblasts Macrophages Neutrophils Hepatocytes	Promotes re-epithelialization Stimulates fibroblast proliferation	[48] [49]
FGF family			
bFGF	Fibroblasts Macrophages Endothelial cells	Acts as a mitogen for fibroblasts Induces angiogenesis Stimulates granulation tissue formation, matrix remodeling, and re-epithelialization	[50] [51]
KGF	Fibroblasts	Acts as a mitogen for epithelial cells	[12]
TGF-β family			
TGF-β1‒3	Platelets Fibroblasts Macrophages Keratinocytes	Acts as a potent chemoattractant for macrophages Acts as a mitogen for fibroblasts Stimulates or inhibits proliferation of various cells Promotes granulation tissue formation and its tensile strength	[37] [46] [52]

**Table 2:** Growth factor (GF) DDSs for wound healing and skin regeneration.

DDS	Method	GF	In Vitro Model	In Vivo Wound Model	Ref.
Liposome	Film formation	EGF		Rat (burn)	[91]
SLN, NLC	Emulsification-ultrasonication	EGF	Human fibroblasts Human keratinocytes	db/db mouse (full-thickness skin excision)	[92]
NLC	Emulsification-ultrasonication	EGF		White pig (full-thickness skin excision)	[93]
PLGA microsphere	W/O/W extraction-evaporation	EGF	Human fibroblasts	Rat (diabetic ulcer)	[94]
PLGA nanoparticle	Double emulsion	EGF	Human fibroblasts	Diabetic rat (full-thickness excision)	[95]
PLGA-alginate microsphere	W/O/W double emulsion-solvent evaporation	EGF		Diabetic rat (full-thickness excision)	[96]
Alginate microsphere	Ion exchange	VEGF		Rat (angiogenesis, small incision in the groin)	[97]
Hyaluronic acid and collagen sponge	Freeze drying	EGF bFGF	Human fibroblasts	db/db mouse (full-thickness dorsal skin excision) Rat (full-thickness abdominal skin excision)	[98] [99] [100]
Chitosan film	Freeze drying	bFGF		db/db mouse (full-thickness dorsal skin excision)	[60]
Chitosan film	Casting	EGF		White pig (full-thickness skin excision)	[101]
Poly(ethylene glycol)-poly(dl-lactide) microfiber	Emulsification electrospinning	bFGF	Mouse embryo fibroblasts	Diabetic rat (full-thickness dorsal skin excision)	[102]
Silk film Silk nanofiber	Casting Electrospinning	EGF		Balb/C mouse (full-thickness dorsal skin excision)	[103]
PLGA nanofiber	Electrospinning	EGF	BALB/c/3T3 A31 fibroblasts	db/db mouse (full-thickness skin excision)	[104]
Gelatin/poly(l-lactic acid)-co-poly-(ε-caprolactone) nanofiber	Electrospinning	EGF	Human fibroblasts Adipose-derived stem cells		[105]
Poly(ε-caprolactone)/poly(ethyleneglycol) nanofiber	Electrospinning	EGF	Human keratinocytes	Streptozotocin-induced diabetic C57BL/6 mouse (dorsal burn)	[106]
Regranex^®^ (carboxymethylcellulose hydrogel)	Mixing	PDGF		Patients with nonhealing and lower extremity diabetic ulcer	[107]
Chitosan gel	Mixing	EGF		Rat (dorsal burn)	[108] [109]
Gelatin gel	Mixing Cross-linking	Platelet-rich fibrin extract		Rat (full-thickness dorsal skin excision)	[110]
Thiol-modified chondroitin 6-sulfate/heparin hydrogel film	Mixing Casting	bFGF		db/db mouse (full-thickness dorsal skin excision)	[111]
Pluronic/chitosan hydrogel	Mixing Cross-linking	EGF	Human fibroblasts	Streptozotocin-induced diabetic C57BL/6 mouse (dorsal burn)	[112]
Heparin/poly(ethylene argininylaspartate digylceride) matrix	Coacervation	FGF-2		C57BL/6 mouse (full-thickness dorsal skin excision)	[113]
HA-EGF conjugate	Coupling reaction between aldehydes and amines	EGF	BALB/c/3T3 cell Human fibroblasts	Rat (full-thickness dorsal skin excision)	[114]
Transactivator of transcription protein-aFGF fusion protein (TAT-aFGF) carbopol gel	Mixing	TAT-aFGF	Human fibroblasts	Rat (pressure ulcer in the greater trochanter)	[115]
Low-molecular weight protamine-EGF conjugate	Gene transfection	EGF	Mouse fibroblasts	Hairless mouse (dorsal burn)	[116] [117]
